# 3-h drain clamping is not effective to reduce total blood loss after primary total knowledge

**DOI:** 10.1186/s43019-020-00051-6

**Published:** 2020-06-19

**Authors:** Dojoon Park, Youn Ho Choi, Kwang Hyun Cho, Hae Seok Koh

**Affiliations:** The Department of Orthopedic Surgery, St. Vincent’s Hospital, The Catholic University of Korea, 93, Jungbu-daero, Paldal-gu, Suwon-si, Gyeonggi-do South Korea

**Keywords:** Total knee arthroplasty, Clamping, Transfusion, Bleeding

## Abstract

**Purpose:**

Total knee arthroplasty (TKA) is a clinically efficacious surgical option for end-stage knee osteoarthritis. However, TKA increases the risk of serious bleeding and blood transfusion. The objective of this study was to evaluate the difference in postoperative blood loss in groups subjected to 3 h of clamping and non-clamping and determine the variations in rate and amount of transfusion after TKA between the two groups.

**Materials and methods:**

Propensity score matching of the group subjected to 3-h drain clamping (43 patients; September 2015 to April 2016) and the control group (43 patients; before initiating the clamping method) was performed in patients undergoing unilateral primary posterior stabilized TKA. The two groups were compared. We measured the total drained blood volume until the drain was removed 48 h after surgery, and we compared the preoperative levels of hemoglobin and hematocrit with levels observed on days 1 and 2 after surgery. We also determined the blood transfusion rate and volume as well as the occurrence of clamping-associated complications.

**Results:**

In the group subjected to 3-h drain clamping, the mean volume of total drained blood was significantly lower than in the control group (333.8 ± 190.2 mL vs. 839.9 ± 339.8 mL, *P* <0.001). There was no significant difference in total blood loss between the two groups (1226.9 ± 488.1 mL vs. 1127.1 ± 424.5 mL, *P* = 0.315), but the hidden blood loss was significantly higher in the 3-h drain clamping group than in the control group (893.1 ± 487.7 mL vs. 294.7 ± 531.8 mL, *P* <0.001). Both the transfusion rate and amount in the 3-h drain clamped group were higher than in the control group but were not statistically significant (30.2% vs. 37.2%, *P* = 0.494 and 269.8 ± 483.8 mL vs. 316.3 ± 158.2 mL, *P* = 0.648, respectively). No significant differences in complications, including deep vein thrombosis, pulmonary thromboembolism, and oozing, were noted between the two groups (all, *P* = 1.000).

**Conclusions:**

The 3-h drain clamping method after primary TKA using posterior stabilized implant reduced the loss of postoperative drained blood. However, hidden blood loss was significantly higher in the 3-h drain clamping group; as a result, there were no differences in total blood loss and transfusion rate. The clamping method did not significantly alter the complication rate.

## Introduction

Total knee arthroplasty (TKA) is one of the most successful orthopedic procedures. However, a significant amount of blood loss and increased rates (up to 39%) of blood transfusion have also been reported [1–3]. Because of blood shortage and the risk of infectious disease transmission and extended hospital stay, surgeons focused on issues related to blood loss and transfusion [4]. Various perioperative blood management strategies, including temporary drain clamping, have been proposed to reduce blood loss and transfusion rates.

Traditionally, many surgeons have preferred negative pressure drainage because of the effectiveness of decreased postoperative pain and edema following hematoma reduction [4]. However, several studies have reported that owing to increased transfusion rates and blood loss, negative pressure drainage after TKA is not advantageous [5–7]. Therefore, surgeons use drains but reduce blood loss by inducing a tamponade with temporary drainage clamping up to a few hours after surgery.

Advocates of temporary drain clamping argue that it reduces blood loss [8–11] because most blood loss after TKA occurs early in the postoperative period (37% in 2 h, 49% in 3 h, and 55% in 4 h) [8]. Although studies have been conducted to confirm the effectiveness of this procedure, most of them used a clamp for 2 or 4 h without any consensus. Furthermore, several perioperative blood conservation strategies, including tranexamic acid or epinephrine injections, have recently been used widely and adopted in a number of studies mentioned above. Therefore, it is not easy to clinically identify the exclusive effect of temporary clamping on blood conservation.

The purposes of this study were to (1) evaluate the possible differences in postoperative blood loss in groups subjected to 3-h clamping and non-clamping, (2) determine whether the rate and amount of transfusion after TKA differed between the two groups, and (3) investigate the differences in complication rates between the two groups.

## Materials and methods

After obtaining ethical approval from the institutional review board of our hospital, we performed a retrospective review of medical records in the TKA registry at the authors’ institution before May 2016. Before September 2015, only negative pressure drainage was used; after that, a 3-h drain clamping method was implemented. A restrictive transfusion threshold of hemoglobin 7 to 8 g/dL was followed in our institution during all periods. Since our institution used tranexamic acid from May 2016, no other perioperative methods were adopted for blood conservation, except drain clamping from September 2015 to April 2016. During this period, 112 TKAs were performed. Unilateral TKA procedures using the same techniques and protocols were included in the analysis. Forty-two patients—27 cases of bilateral TKA, 4 cases of revision TKA, and 11 patients who had a history of coagulation disorders or anticoagulant treatment—were excluded from the study.

To compensate for selection bias in the observational study, patients included in the non-clamping period were compared with patients in the clamping period using propensity score matching (PSM). Seven clinically relevant variables were chosen for imputation and derivation of propensity scores. Variables were age, sex, body mass index, diagnosis, preoperative anemia, history of aspirin intake, and type of implant. Using PSM, we obtained a well-balanced one-to-one matching to ensure successful matching of all variables. The details of the patients are listed in Table 1. Finally, the data of 86 patients (3-h drain clamping group, *n* = 43; control group, *n* = 43) were included in the analysis.

**Table 1:** Baseline characteristics of enrolled patients

	3-h drain clamping group (*n* = 43)	Control group (*n* = 43)	*P* value
Age, year	71.9 ± 6.5	72.0 ± 6.4	0.756
Sex, male: female	7: 36	7: 36	1.000
Body mass index, kg/m^2^	25.1 ± 3.3	25.2 ± 3.2	0.611
Diagnosis			1.000
Osteoarthritis	41 (95.3%)	41 (95.3%)	
Rheumatoid arthritis	2 (4.7%)	2 (4.7%)	
Preoperative anemia	12 (27.9%)	13 (30.2%)	0.640
Aspirin	7 (16.3%)	5 (11.6%)	0.218
Prosthesis, NexGen LPS: Genesis II	33: 10	33: 10	1.000

Preoperative anemia was diagnosed through a preoperative assessment, and threshold was defined by the World Health Organization (<12.0 g/dL in women and <13.0 g/dL in men)

Values were presented as mean ± standard deviation or as number (percentage)

*Abbreviations*: *Genesis II* posterior stabilized Genesis II TKA (Smith & Nephew, Memphis, TN, USA), *NexGen LPS* NexGen Legacy Posterior Stabilized TKA (Zimmer, Warsaw, Indiana, USA)

All operations were performed by a single experienced surgeon, our senior author. A dose of 1 g cefamezin was injected intravenously before skin incision. The inflation pressure of the pneumatic tourniquet was 300 mm Hg. A medial parapatellar arthrotomy was carried out in all operations. A posterior stabilized implant was used, and the patella was not resurfaced in all cases. Two types of implants were used: NexGen LPS-Flex (Zimmer, Warsaw, IN, USA) and Genesis II (Smith & Nephew, Memphis, TN, USA). An intramedullary alignment guide was used for femoral cutting, and the femoral canal was filled with a bone plug. An extra-medullary guide was used for tibial cutting. Bone cement was used to fix the implants to the femur and tibia. After cementation, the tourniquet was deflated and meticulous hemostasis was performed via electric cauterization. Before joint capsule closure, the suction drain was placed in the intra-articular space. The drain tube and negative pressure drainage bag (400 mL, Barovac, Sewoon Medical, Seoul, Republic of Korea) were connected after joint closure. In all cases included in this analysis, no tranexamic acid or epinephrine was used. In the control group, negative pressure drainage was carried out immediately after connecting the tube and bag. In the test group, clamping was performed while connecting the drainage bag. During the clamping, the time was noted on the bag. Three hours later, the ward physician released the clamp and started negative pressure drainage. All suction drains were removed at 48 h after operation by ward physician, and the amount of drained blood was recorded by ward nurses. Hematologic labs were performed on days 1 and 2 after surgery. After written consent was obtained, the patients received blood transfusion if the hemoglobin level decreased below 7 g/dL with asymptomatic anemia and the hemoglobin level was less than 8 g/dL in patients with cardiovascular disease or anemic symptoms such as dizziness or lightheadedness. To prevent deep vein thrombosis (DVT), an intermittent pneumatic compression device was used in all patients and compression stockings were worn. However, no chemoprophylaxis such as low-molecular-weight heparin or direct Xa inhibitor was administered. The medical records obtained from outpatient and general wards were analyzed to obtain clinical data. In particular, demographic information, primary diagnosis, and history of drug treatment and blood transfusion were investigated. Postoperative blood loss was evaluated and analyzed as follows. The total drained blood (TDB) was obtained by the total sum of the drained blood until the drain was removed. Total blood loss was assumed to be estimated blood loss (EBL) calculated by using the Gross formula:

$$ \mathrm{EBL}=\mathrm{BV}\ \left[\mathrm{Hct}\ \left(\mathrm{i}\right)\ \mathrm{hct}\ \left(\mathrm{f}\right)\right]/\mathrm{Hct}\ \left(\mathrm{m}\right)\Big], $$

where BV denotes the blood volume, calculated as a product of body weight (kg) and 70 mL/kg; Hct (i), Hct (f), and Hct (m) are the initial, final, and mean (of the initial and final) hematocrit levels, respectively [12]. Hidden blood loss is calculated by subtracting TDB from EBL, which includes residual blood in the joint, extravasated blood, and losses due to hemolysis. DVT and pulmonary thromboembolism (PTE) due to clamping of the drainage system were evaluated. DVT screening was routinely performed on postoperative day 7 via Doppler sonography by radiologists, and angiography was performed to evaluate DVT and PTE when the screening result was positive. Oozing was defined when wound dressing material had to be reinforced. Wound infection was assessed until the day of discharge.

### Statistical analysis

Based on data derived from a previous study [13] and analysis of a pilot study, it was confirmed that 43 patients in each group were sufficient for power analysis with a probability of 0.9 to determine the difference between the two groups at a significance level of 0.05. Descriptive statistics were presented as mean ± standard deviation or percentages of participants. Statistical comparison of continuous variables was conducted by using the Student’s *t* test or Mann–Whitney *U* test, whereas categorical variables were analyzed by using the chi-squared test or Fisher’s exact test (as appropriate). *P* values of less than 0.05 were considered statistically significant. The statistical analyses were conducted using Microsoft Excel 2013 (Microsoft, Redmond, WA, USA) and SPSS software (version 20; SPSS Inc., Chicago, IL, USA).

## Results

In the group subjected to drain clamping for 3 h, the mean amount of TDB volume was significantly lower than in the control group (333.8 ± 190.2 mL vs. 839.9 ± 339.8 mL, *P* <0.001). There was no significant difference in total blood loss between the two groups (1226.9 ± 488.1 mL vs. 1127.1 ± 424.5 mL, *P* = 0.315), whereas hidden blood loss was significantly higher in the group subjected to 3-h drain clamping than in the control group (893.1 ± 487.7 mL vs. 294.7 ± 531.8 mL, *P* <0.001). Hemoglobin and hematocrit levels did not vary significantly between the two groups before surgery and 1 to 2 days after surgery (Table 2).

**Table 2:** Blood loss after surgery and hemoglobin and hematocrit levels before and after surgery

	3 h drain clamping group (*n* = 43)	Control group (*n* = 43)	*P* value
Total drained blood, mL	338 ± 190.2	839 ± 339.8	<0.001
Total blood loss, mL	1226.9 ± 488.1	1127.1 ± 424.5	0.315
Hidden blood loss, mL	893.1 ± 487.7	294.7 ± 531.8	<0.001
Hemoglobin, g/dL			
Preoperation	12.7 ± 1.3	13.0 ± 1.3	0.286
Postoperative 1 day	9.7 ± 1.1	10.2 ± 1.5	0.216
Postoperative 2 day	8.9 ± 1.0	9.6 ± 1.4	0.105
Hematocrit, percentage			
Preoperation	36.8 ± 4.1	37.5 ± 3.6	0.397
Postoperative 1 day	28.3 ± 3.3	29.2 ± 3.8	0.243
Postoperative 2 day	26.6 ± 3.3	28.1 ± 3.7	0.061

Normally distributed data given as mean ± standard deviation

Thirty patients in the experimental group and 16 patients in the control group received blood transfusion. Both the transfusion rate and amount in the 3-h drain clamped group were higher than in the control group but were not statistically significant (30.2% vs. 37.2%, *P* = 0.494 and 269.8 ± 483.8 mL vs. 316.3 ± 158.2 mL, *P* = 0.648, respectively). No significant differences in complications including DVT, PTE, and oozing were noted between the two groups (all, *P* = 1.000). No wound infection occurred in any patient (Table 3).

**Table 3:** Transfusion rate, transfusion amounts, and complication rate between the two groups

	3-h drain clamping group (*n* = 43)	Control group (*n* = 43)	*P* value
Transfusion			
Transfusion rate	13 (30.2%)	16 (37.2%)	0.494
Transfusion amount	269.8 ± 483.8	316.3 ± 458.2	0.648
Complications			
Deep vein thrombosis	3 (7.0%)	2 (4.7%)	1.000
Pulmonary thromboembolism	1 (2.3%)	1 (2.3%)	1.000
Oozing	3 (7.0%)	2 (4.7%)	1.000
Wound infection	0	0	

Values were presented as mean ± standard deviation or as number (percentage)

## Discussion

A comparison of patients subjected to 3 h of drain clamping and those who received negative pressure drainage confirmed that drain clamping for 3 h reduced the TDB. However, the hidden blood loss was higher in the group subjected to 3-h drain clamping; as a result, the total blood loss did not differ between the two groups.

Despite the significant decrease in blood loss during surgeries over the past 20 years, blood loss associated with TKA is still a concern. Allogeneic blood transfusion has traditionally been used to treat acute blood loss without analyzing the associated risks and benefits. According to the last annual report of Serious Hazards of Transfusion, 109 patients had a major morbidity and 20 died, including eight cases probably related and 12 possibly related to transfusion [14]. Therefore, several studies have been conducted to determine effective and safe strategies to reduce bleeding. Several perioperative techniques, including temporary drain clamping, treatment with fibrin, and tranexamic acid or epinephrine injections, have been considered to reduce blood loss after TKA. Among the various methods used, temporary drain clamping is the simplest and most cost-effective.

Since most blood loss after TKA occurs early after surgery [8–11], it is assumed that temporary clamping of the drainage tube for the first few hours after TKA can reduce the bleeding via a tamponade effect. Several studies have demonstrated the effect of drain clamping in reducing blood loss and the need for transfusion [9, 10, 15–18]. One of the most important issues underlying drain clamping is the timing of release. Releasing the clamp too fast will not generate adequate tamponade effect, which results in insufficient bleeding control. Prolonged clamping, however, can lead to complications such as swelling and hematoma, which can trigger pain or infection. However, no consensus has been reached to establish the optimal clamping duration to maximize the effect of bleeding control and minimize other adverse effects. Studies have been conducted to determine the optimum clamping duration. According to a study by Kiely et al. [9], 2 h of drain clamping was not effective. Stucinskas et al. [11] demonstrated that 4 h of drain clamping significantly reduced postoperative blood loss and diminished the frequency and volume of blood transfusion but not significantly. In another study, Jung et al. [19] reported no difference in total blood loss, hemoglobin, or hematocrit between negative pressure drainage and 4-h drain clamping with an epinephrine injection. However, a significantly higher number of wound-related challenges occurred in the clamping group. According to Jeon et al. [13], clamping for both 3 h and 4 h was effective in reducing the drained volume compared with no clamping when all patients received hemocoagulase intravenously before the operation. In our study, the TDB decreased in the group exposed to 3 h of drain clamping; however, due to the increased level of occult blood loss in the clamped group, no difference existed in the total blood loss.

In general, the volume of drained blood is the most intuitive clinical indicator of postoperative blood loss. However, because of residual blood after TKA within the joint in the form of hematoma or absorption into the surrounding soft tissues and leakage through the wound, the drained blood does not represent the entire postoperative bleeding event [19]. Sehat et al. also reported that substantial hidden blood loss in TKA was not uncommon [20]. Clamping for 3 h significantly increased hidden blood loss compared with negative pressure drainage. Thus, despite the decrease in TDB via intermittent clamping, the levels of postoperative hemoglobin and hematocrit in the clamped group did not differ from those of the control group.

One of the ultimate goals of all blood conservation methods is to reduce the rate of blood transfusion. Tai et al. [21, 22] systematically reviewed the literature, and subsequent meta-analysis of randomized controlled trials did not result in adequate evidence to support temporary drainage clamping after TKA. A review of randomized controlled trials comparing all types of temporary or non-clamping drainage showed that patients who were exposed to temporary drainage clamping for 4 h or more had a higher hemoglobin level 24 h postoperatively than those who were not exposed to clamping, and the number of blood transfusions per patient decreased significantly [23]. In our present study, the 3-h clamping reduced blood transfusion rate compared with the control group, but the difference was not statistically significant. Therefore, the findings suggest a failure of patients exposed to 3-h clamping to show higher postoperative hemoglobin and hematocrit levels postoperatively on days 1 and 2 compared with the control group.

Intermittent drain clamping was adopted to minimize blood loss via tamponade effect and was released at appropriate intervals to minimize hematoma formation. Owing to concerns related to the timing of release and the resulting side effects, previous studies investigated the increased risk of DVT or wound-related complications, including oozing and infection. Huang et al. [23] analyzed 703 knees in seven trials and reported no difference in the incidence of wound-related complications between clamping and control groups. Analysis of DVT in six trials revealed no difference in the prevalence of DVT. Our study revealed no difference in the occurrence of DVT, PTE, and oozing between the two groups. No wound infection was detected in any patient.

This study has the following limitations. First, there was inherent selection bias due to the retrospective nature of the study. We tried to overcome the limitation using PSM techniques to perform a well-matched and balanced comparison based on clinical factors that affected the results. Second, the number of patients in the study group was limited. This study confirmed the effect of clamping alone on blood conservation without using additional methods, such as tranexamic acid. We selected 43 patients as the study group subjected to clamping on the basis of the inclusion and exclusion criteria, specifically for short periods. The limited statistical power due to small patient numbers may have contributed to statistically insignificant comparisons. However, given the power analysis based on previous studies, the expansion of sample size is not expected to significantly change our findings. Third, intraoperative factors that cause potential bleeding, such as operation time, extended bone cut, or soft tissue release, were not considered. However, the fact that a high-volume single surgeon performed all operations would have controlled intraoperative confounders.

## Conclusions

The 3-h drain clamping method after primary posterior stabilized TKA reduced postoperative loss of drained blood. However, the hidden blood loss was significantly higher in patients exposed to 3-h drain clamping; as a result, there were no differences in total blood loss and transfusion rate; the 3-h drain clamping method did not significantly alter the rates of complication. Further studies investigating the role of clamping in reducing postoperative blood loss are needed using a combination of methods such as tranexamic acid and clamping time adjustment.

## Data Availability

The datasets used or analyzed (or both) in this study are available from the corresponding author upon reasonable request.

## Abbreviations

DVT: Deep vein thrombosis
EBL: Estimated blood loss
Hct: Hematocrit
PSM: Propensity score matching
PTE: Pulmonary thromboembolism
TDB: Total drained blood
TKA: Total knee arthroplasty

## References

[1] Ritter MA, Keating EM, Faris PM (1994). Closed wound drainage in total hip or total knee replacement. A prospective, randomized study. J Bone Joint Surg Am.

[2] Lotke PA, Faralli VJ, Orenstein EM, Ecker ML (1991). Blood loss after total knee replacement. Effects of tourniquet release and continuous passive motion. J Bone Joint Surg Am.

[3] Callaghan JJ, Spitzer A (2000). Blood management and patient specific transfusion options in total joint replacement surgery. Iowa Orthop J.

[4] Canty SJ, Shepard GJ, Ryan WG, Banks AJ (2003). Do we practice evidence based medicine with regard to drain usage in knee arthroplasty? Results of a questionnaire of BASK members. Knee.

[5] Esler C, Blakeway C, Fiddian N. The use of a closed-suction drain in total knee arthroplasty: a prospective, randomised study. J Bone Joint Surg (Br) 2003;85(2):215–217. 10.1302/0301-620x.85b2.13357 12678355

[6] Niskanen R, Niskanen RO, Korkala OL, Haapala J, Kuokkanen HO, Kaukonen JP, Salo SA (2000). Drainage is of no use in primary uncomplicated cemented hip and knee arthroplasty for osteoarthritis: a prospective randomized study. J Arthroplast.

[7] Parker MJ, Roberts CP, Hay D. Closed suction drainage for hip and knee arthroplasty: a meta-analysis. J Bone Joint Surg Am 2004;86(6):1146–1152. 10.2106/00004623-200406000-00005 15173286

[8] Senthil Kumar G, Von Arx OA, Pozo JL (2005). Rate of blood loss over 48 hours following total knee replacement. Knee.

[9] Kiely N, Hockings M, Gambhir A (2001). Does temporary clamping of drains following knee arthroplasty reduce blood loss? A randomised controlled trial. Knee.

[10] Shen P-C, Jou I-M, Lin Y-T, Lai K-A, Yang C-Y, Chern T-C (2005). Comparison between 4 hour clamping drainage and nonclamping drainage after total knee arthroplasty. J Arthroplast.

[11] Stucinskas J, Tarasevicius S, Cebatorius A, Robertsson O, Smailys A, Wingstrand H (2009). Conventional drainage versus four hour clamping drainage after total knee arthroplasty in severe osteoarthritis: a prospective, randomised trial. Int Orthop.

[12] Gross JB (1983). Estimating allowable blood loss: corrected for dilution. Anesthesiology.

[13] Jeon YS, Park JS, Kim MK (2017). Optimal release timing of temporary drain clamping after total knee arthroplasty. J Orthop Surg Res.

[14] Frietsch T (2019). Research for transfusion safety- priority of administration safety. Transfus Apher Sci.

[15] Aksoy Y, Altinel L, Köse K (2011). The comparison of the effects of intraoperative bleeding control and postoperative drain clamping methods on the postoperative blood loss and the need for transfusion following total knee arthroplasty. Acta Orthop Traumatol Turc.

[16] Madadi F, Mehrvarz A, Madadi F, Boreiri M, Abachizadeh K, Ershadi A (2010). Comparison of drain clamp after bilateral total knee arthroplasty. J Knee Surg.

[17] Prasad N, Padmanabhan V, Mullaji A (2005). Comparison between two methods of drain clamping after total knee arthroplasty. Arch Orthop Trauma Surg.

[18] Sa-ngasoongsong P, Sa-ngasoongsong P, Channoom T, Kawinwonggowit V, Woratanarat P, Chanplakorn P, Wibulpolprasert B et al (2011) Postoperative blood loss reduction in computer-assisted surgery total knee replacement by low dose intra-articular tranexamic acid injection together with 2 hour clamp drain: a prospective triple-blinded randomized controlled trial. Orthop Rev 3(2) 10.4081/or.2011.e12 PMC3206515 22053253

[19] Jung WH, Chun C-W, Lee J-H, Ha J-H, Kim J-H, Jeong J-H (2013). No difference in total blood loss, haemoglobin and haematocrit between continues and intermittent wound drainage after total knee arthroplasty. Knee Surg Sports Traumatol Arthrosc.

[20] Sehat K, Evans R, Newman J (2004). Hidden blood loss following hip and knee arthroplasty: correct management of blood loss should take hidden loss into account. J Bone Joint Surg (Br).

[21] Tai T-W, Yang C-Y, Jou I-M, Lai K-A, Chen C-H (2010). Temporary drainage clamping after total knee arthroplasty: a meta-analysis of randomized controlled trials. J Arthroplast.

[22] Tai TW, Jou I-M, Chang C-W, Lai K-A, Lin C-J, Yang C-Y (2010) Non-drainage is better than 4 hour clamping drainage in total knee arthroplasty. Orthopedics 33(3) 10.3928/01477447-20100129-11 20349865

[23] Huang Z, Ma J, Pei F, Yang J, Zhou Z, Kang P, Shen B (2013). Meta-analysis of temporary versus no clamping in TKA. Orthopedics.

